# Baltimore College of Dental Surgery

**Published:** 1892-04

**Authors:** 


**BALTIMORE COLLEGE OF DENTAL SURGERY.**—The fifty-second annual commencement exercises of the Baltimore College of Dental Surgery were held at the Lyceum Theater, Baltimore, Md., on Monday evening, March 21, 1892.

The annual oration was delivered by the Rev. F. M. Ellis, and the valedictory by Philip Ernest Sasscer, D. D. S.

The number of matriculates for the session was one hundred and eighty-one.

The degree of D.D.S. was conferred on the following graduates by Professor R. B. Winder, dean of the faculty:

Benjamin D. Altemus, Pennsylvania; Charles W. Arird, Pennsylvania; John N. Baker, Pennsylvania; Irwin J. Beach, Maryland; William J. Beatty, Pennsylvania; Charles A. Bland, North Carolina; Charles W. Boucher, Maryland; H. V. Bradshaw, Pennsylvania; B. Bridgforth, Virginia; Burt B. Brumbaugh, Pennsylvania; James C. Buchanan, Pennsylvania; William C. Callahan, New York; Walter C. Carter, Missouri; F. A. Charles, Massachusetts; Charles A. Cochel, Maryland; Robert S. Cole, North Carolina; Edwin Davis, Pennsylvania; Willey C. Dawson, West Virginia; Jacob W. Derlin. Maryland; J. S. Donaldson, D.D.S., Colorado; J. R. D'onaldson, D.D.S., Colorado; Harry Donnan, Pennsylvania; Benj. F. Dulaney, Texas; Nelson H.Ehle, Minnesota; Mortimer L. Fay, New York; Howard R. Fonda, Vermont; Harley B. Ford, Canada; Fred. A. Ford, New York; Alexander Francis,Maryland; Isaac A.Frazer, California; John N. Giddens,Alabama; Richard L. Gill, Maryland; W.I. Goodwin, Canada; Harry W.Graham, Pennsylvania; Archer C.Griffith,California; E.Grosheintz, D.D.S., Switzerland; Clarkson N. Guyer, Colorado; George F. Hair, South Carolina; Charles E. Hamilton, Georgia; W. I. Hatch, B. A., South Carolina; Julio Hidalgo, Venezuela; W. S. Holbrook, New Jersey; Frank H. Jackman, Connecticut; A. Jekelfalhsy, Wisconsin; George M. Jones, Iowa; Earnest P. Keerans, North Carolina; Clinton Kenney, Connecticut; F. H. Kestler, California; Edward T. Ketcham, California; Harry W.Knauff,Pennsylvania; Milo D. Kottraba; Robert M. Krebs, Pennsylvania; Joseph E. La Force, Oregon; E. T. H. Leonard, Mississippi; James I. Logan, Alabama; William S. Long, North Carolina; William L. Lowe, Pennsylvania; H. H. Maloney, A.M., Louisiana; Edgar W. Marven, Canada; James W. Moore, Canada; Simon B. Meyer, Maryland; Patrick McCabe, Australia; Charles C. McCloud, Louisiana; George B. McFarland, East Indies; William M. McGraw, Pennsylvania; Peter A. McLean, New Jersey; Ellis MacDougall, New York; John E. Parker, Texas; Leo Arthur Pusey, Virginia; Edgar K. Rainey, Georgia; Louis A. Reinhart, Maryland; Isaac L. Ritter, Pennsylvania; Robert I. Robertson, Canada; Ryland O.Sadler, North Carolina; Philip E. Sasscer, Maryland; George H. Sayre, New York; A. S. Shackleford, Texas; Zadoc P. Shaw, Maine; John H. Smith, Virginia; William H. Stokes, New York; John E. Storey, Texas;

James T. Stuart, Alabama; Fred. W. Sweezy, New York; William A. Taylor, Maryland; Fred. A. Taylor, Canada; Clarence H. Terry, Texas; William P. Terry, Louisiana; Albert G. Tillman, Mississippi; Eduardo Vasquez, Guatemala; Henry A. Truxillo, Louisiana; William H. Waters, Maryland; Thomas F. Warnes, New York; Joseph D. Whiteman, Pennsylvania; Edgar L. Wilder, Vermont; Benj. H. Williams, Georgia; David M. Wilson, New York; Charlie H. Winburn, Georgia; James I. Woolverton, New Jersey; Frederio W. Wright, Canada; James A. Yates, Kentucky; Robert I. Youngs, New York; Rudolph L. Zelenka, Louisiana.

